# Intussusception of the vermiform appendix caused by mucinous tumor of the appendix: Case report

**DOI:** 10.1016/j.ijscr.2020.01.024

**Published:** 2020-01-27

**Authors:** Dildar Haji Musa, Ayad Ahmad Mohammed

**Affiliations:** Department of Surgery, College of Medicine, University of Duhok, Kurdistan Region, Iraq

**Keywords:** Intussusception, Right iliac fossa pain, Acute appendicitis, Appendicectomy, Mucinous cystadenoma

## Abstract

**Introduction:**

Intussusception of the appendix occurs when part of the appendix passes the part next to it or the whole appendix to the cecum. It is classified as primary or secondary, or can be classified to partial or complete. Most cases are secondary and partial. Patients usually presents with right lower abdominal pain and most cases are diagnosed during surgery.

**Case presentation:**

A 27-year-old female presented with right iliac fossa pain and nausea for 2 days. Abdominal examination showed tenderness, guarding, and rebound tenderness at the right iliac fossa. The WBC count was 11,000 c/mm and the urinalysis was normal.

At surgery, there was intussusception of the vermiform appendix in its middle part with palpable mass attached to its wall. Appendicectomy was performed successfully. The result of the histopathology was consistent with mucinous cystadenoma of the appendix with no evidence of malignancy.

**Conclusion:**

Surgery is the main form of treatment as most of the cases are diagnosed during surgery, the operation type may include appendicectomy or more extensive surgery is required such as right hemicolectomy depending on the cause. Surgery can be performed laparoscopically. Trials of colonoscopic reduction are not recommended.

## Highlights

• Acute appendicitis is the commonest surgical emergency worldwide.
• Surgery is usually needed which could be done by open or laparoscopic techniques.
• Tumors of the appendix may cause intussusception of the appendix.

## 1 Introduction

Intussusception of the appendix is defined as invagination of part of the vermiform appendix to the part next to it or the whole appendix inside the cecum. The condition may be primary and more common in young age groups but cases have been reported affecting infants and elderly patients, males are affected more commonly than females, and most cases are secondary. It may be partial involving part of the appendix or complete in which the whole appendix passes to the cecum [1,2].

In primary cases no causative agent can be detected, but the secondary type occurs when there is a causative pathological condition such as inflammatory conditions, tumors of the vermiform appendix, the presence of faecalith inside the lumen of the appendix, endometriosis involving the appendix, or some other rare pathological factors [3].

Patients usually present with symptoms of appendicitis like right lower abdominal pain which varies in intensity and duration, in some patients the patients present with severe pain requiring an emergency operation, while others may have chronic and recurrent attacks of pain with multiple hospital visits before the condition is diagnosed, patients may also have shifting pain, nausea, vomiting and fever. Many cases are diagnosed at autopsy [2,4,5].

The condition is mostly diagnosed during surgery, sometimes and in some rare occasions the condition may be diagnosed radiologically preoperatively, CT-scan is the most useful diagnostic tool, the characteristic radiological sign is the coiled-spring appearance of the cecum with no filling of the appendix lumen when the double-contrast barium enema is used. Colonoscopy when performed may show the invaginated appendix inside the lumen of the cecum, this finding is seen in the complete type, while it may be completely normal in the partial type. The differential diagnoses may include tumors of the vermiform appendix and tumors of the cecum [6,7].

The work of this report case has been reported in line with the SCARE 2018 criteria [8].

## 2 Patient information

A 27-year-old female was referred to the emergency department complaining from right iliac fossa pain and nausea for the last 2 days.

The patient has negative past medical and surgical histories, and the family history was negative for chronic illnesses.

### 2.1 Clinical findings

The general examination was unremarkable. Abdominal examination showed tenderness, guarding, and rebound tenderness at the right iliac fossa.

### 2.2 Diagnostic assessment

The WBC count was 11,000 c/mm and the urinalysis was normal. Ultrasound of the abdomen showed a simple right ovarian cyst measuring 3 cm in diameter with no evidence of pelvic collection.

### 2.3 Therapeutic intervention

**Fig. 1:**
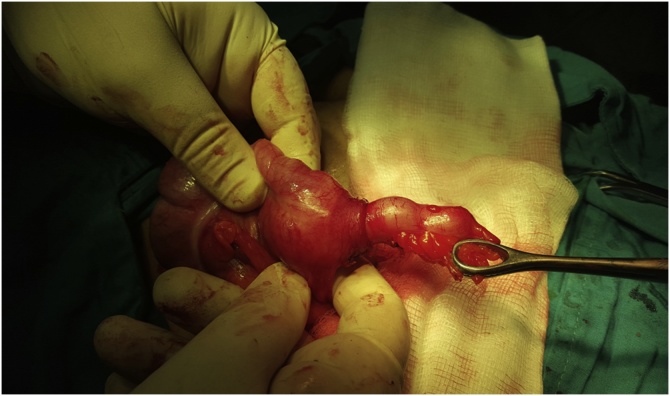
An intraoperative picture showing the invaginated vermiform appendix.

Decision for Appendicectomy was done, during surgery the vermiform appendix was invaginated in its middle part with palpable mass attached to its wall, Fig. 1.

**Fig. 2:**
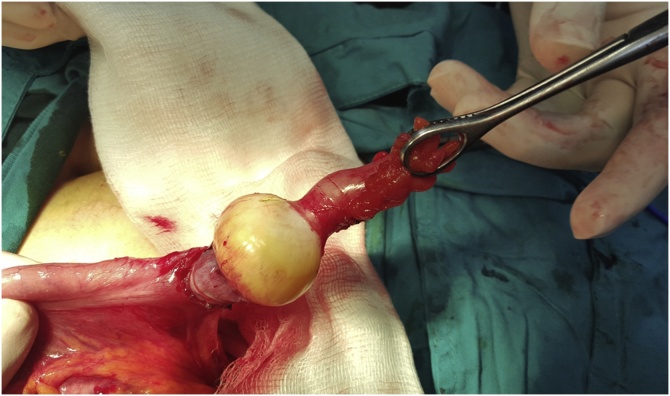
An intraoperative picture showing a round yellowish tumor in the middle part of the vermiform appendix which was the lead point of the intussusception.

Traction applied to the tip of the appendix and there was round yellowish mass that was causing this invagination or interception, Fig. 2.

Appendicectomy was performed successfully and the right ovarian cyst removed.

The result of the histopathology was consistent with mucinous cystadenoma of the appendix with no evidence of malignancy.

### 2.4 Follow-up and outcomes

The patient was admitted for 2 days with no postoperative complications.

## 3 Discussion

The first reported case of appendiceal intussusception was in 1858, when McKidd reported the condition in an autopsy sample of seven-year-old child who had history of repeated attacks of colicky abdominal pain 2 months before his death. This case was associated with round worm infection and was regarded as secondary. Appendiceal intussusception may be primary in which there is an invagination of the appendix but more commonly it is secondary which is caused by a predisposing cause [1].

After the first reported case of McKidd, in 1897, Wright and Renshaw reported the first case who was diagnosed during surgery and underwent a successful surgery with appendicectomy [1].

Most cases are caused by tumors of the appendix and endometriosis, mucocele of the appendix, carcinoid tumor, adenomas, and adenocarcinoma have been reported to be the causative tumors in many reported cases [2].

Compound ileo-cecal or ileo-ceco-colic intussusception may be found in association with appendiceal intussusception in approximately half of the affected patients, this may suggest that this may be the triggering step in such patients [1].

The coiled-spring appearance in CT-scan may be seen after appendicectomy when the stump of the appendix invaginates inside the cecum [6].

In some old case reports successful hydrostatic pressure had been done for some cases with good long term outcome, but this is performed for the primary cases and when the patient is unwilling to have the surgical option. In many of the recent publications any authors [2].

Surgery is the main form of treatment as most of the cases are diagnosed during surgery, the operation type may include appendicectomy when the condition is primary or the causative agent is benign, some times more extensive surgery is required such as right hemicolectomy when there is a malignant tumors causing the intussusception. Surgery may be done either by the open technique or the laparoscopic one, trials of colonoscopic reduction are not recommended by most of the authors as it may be very difficult and may result in some serious complications or the condition may be secondary and the primary lesion is missed which results ln delayed presentation [7,9].

### 3.1 Patient’s perspective

I was worried about the results of the surgery and after that my doctor informed me that this is a benign condition and no further act is required.

## Funding

None.

## Ethical approval

Ethical approval has been exempted by my institution for reporting this case.

## Consent

Written informed consent was obtained from the patient for publication of this case report and accompanying images.

## Author contribution

The concept of reporting the case, data recording is done by Dr Dildar Haji Musa and Dr Ayad Ahmad Mohammed.

Drafting the work and final approval of the work to be published is done by Dr Ayad Ahmad Mohammed.

## Registration of research studies

This work is case report and there is no need of registration.

## Guarantor

Dr Ayad Ahmad Mohammed is guarantor for the work.

## Provenance and peer review

Not commissioned, externally peer-reviewed.

## Declaration of Competing Interest

The author has no conflicts of interest to declare.

## References

[1] Langsam L.B., Raj P.K., Galang C.F. (1984). Intussusception of the appendix. Dis. Colon Rectum.

[2] Kleinman P. (1980). Intussusception of the appendix: hydrostatic reduction. Am. J. Roentgenol..

[3] Sakaguchi N. (1995). Intussusception of the appendix: a report of three cases with different clinical and pathologic features. Pathol. Int..

[4] Kimura H. (1999). Intussusception of a mucocele of the appendix secondary to an obstruction by endometriosis: report of a case. Surg. Today.

[5] Arif S.H., Mohammed A.A. (2019). Gangrenous vermiform appendix inside a strangulated inguinal hernia in an infant; a rare variety of Amyand’s hernia. J. Pediatr. Surg. Case Rep..

[6] Levine M. (1985). Coiled-spring sign of appendiceal intussusception. Radiology.

[7] Laalim S.A. (2012). Appendiceal intussusception to the cecum caused by mucocele of the appendix: laparoscopic approach. Int. J. Surg. Case Rep..

[8] Agha R.A. (2018). The SCARE 2018 statement: updating consensus Surgical CAse REport (SCARE) guidelines. Int. J. Surg..

[9] Mohammed A.A., Ghazi D.Y., Arif S.H. (2019). Ingested metallic foreign body impacted in the vermiform appendix presenting as acute appendicitis: case report. Int. J. Surg. Case Rep..

